# Surgical limb‐sparing in veterinary medicine: A review of existing techniques in dogs

**DOI:** 10.1111/vsu.70072

**Published:** 2025-12-25

**Authors:** Johnny Altwal, Bernard Séguin

**Affiliations:** ^1^ College of Veterinary Medicine and Biomedical Sciences, Colorado State University Fort Collins Colorado USA; ^2^ Department of Clinical Sciences College of Veterinary Medicine, Cornell University Ithaca New York USA; ^3^ Central Victoria Veterinary Hospital Victoria British Columbia Canada

## Abstract

Surgical limb‐sparing in veterinary medicine can be defined as an intervention aimed at preserving limb function when a bone abnormality is present, namely neoplasia or a non‐repairable fracture, and the affected segment of that bone needs to be removed and, most often, replaced. In some cases, the affected segment of bone is treated and reimplanted. It is mostly prevalent in the context of local tumor control while preserving limb function in veterinary surgical oncology but has also been employed for comminuted fracture repair. Importantly, this review focuses on neoplasia and non‐repairable fractures wherein the bones were normal prior to the pathology and the non‐affected segments of bone remain normal in the face of the pathology. Several techniques have been reported and vary based on a number of factors such as anatomic location of the pathology and method of addressing the defect created by removal of the affected bone segment. Limb‐sparing techniques have been documented to have comparable survival times to limb amputation but can be fraught with mechanical and biological complications, requiring intensive long‐term care and client compliance with treatment regimens. The most common complications are infection, mechanical failure, and local recurrence. Decreasing the risk of complication is the driving force for research in the field of limb sparing in dogs. The aim of this review was to compile the existing literature on surgical limb‐sparing in dogs with the intent to guide clinical decision‐making and inform further research on limb‐sparing.

## 1 INTRODUCTION

Before surgical limb‐sparing was developed, limb amputation was the only surgical procedure available to small animals for the treatment of appendicular skeletal neoplasia or traumatic bone injury in a limb not amenable to surgical stabilization.^1^ Limb amputations are relatively common in veterinary medicine with generally low morbidity and mortality, and good to excellent functional outcomes in dogs of all sizes, albeit there is a paucity of objective data regarding outcome after amputation in dogs.^1^, ^2^, ^3^, ^4^ Despite this, some clients may oppose limb amputation. One study found that 50% of respondents expressed serious objections to amputation for their pet.^4^ The majority of concerns expressed were considered emotional by the investigators, surrounding the pet's appearance and the perceptions others will have of the dog postoperatively.^4^ Additionally, 20% of owners reported a decrease in their dog's recreational activities after amputation, 38% reported a decrease in their dog's stamina during exercise, and 32% reported behavioral changes such as increased aggression toward other dogs, increased anxiety, decreased dominance, and lack of interest in other dogs.^2^, ^4^ Despite such concerns, the majority of owners found that their dog had good quality of life post‐amputation.^2^, ^4^ Limb amputations are mainly contraindicated for severely obese dogs as well as those with significant orthopedic or neurologic comorbidities affecting other limbs.^1^, ^4^

Surgical limb‐sparing provides clients and their pets an alternative to amputation. Stated simply, limb‐sparing is treating bone pathology in a way that preserves limb function. The term most often refers to addressing a pathology that leads to a critical bone defect. This is in contrast to a traumatic fracture that is repaired, that is, stabilized. A critical bone defect is defined as a defect or gap in the bone caused by bone loss that would not heal spontaneously despite surgical stabilization and requires further surgical intervention, such as autologous bone grafting.^5^, ^6^ Defects larger than 2 cm in length and with more than 50% circumferential bone loss are considered critical bone defects in humans.^7^

The term limb‐sparing is typically used for interventions that will not rely on the use of an exoprosthesis which is considered a partial amputation when used. The authors acknowledge that some techniques rely on a prosthesis that is a hybrid between an exo‐ and endoprosthesis, such as intraosseous transcutaneous amputation prosthesis (ITAP),^8^, ^9^ which can be considered a combination between a partial amputation and surgical limb‐sparing surgery. The present review focuses on bone neoplasia and severe fractures based on the premise that the limb is presumed to have been normal prior to the pathology and the non‐affected segments of bone remain normal after the onset of the pathology in such cases. Limb‐sparing takes many forms, and the different techniques can be organized based on the technical approach used to treat the bone pathology while maintaining limb function (Table 1). Table 2 organizes limb‐sparing techniques based on the most common osteosarcoma (OSA) sites they have been used in.

**TABLE 1:** Organization of the different surgical limb‐sparing techniques used clinically in dogs based on technical approach.

Technical approach	Limb‐sparing techniques		
Limb reconstructed (section 2.1)	Cortical allograft (section 2.1.1)		
	Endoprosthesis implant (section 2.1.2)	Metal (section 2.1.2.1)	Off the shelf/commercially available (section 2.1.2.1.1)
			Patient‐specific 3D‐printed (section 2.1.2.1.2) (can also include hybrid exo‐/endo‐prosthesis for ITAP)
		Bioreplaceable Implants (section 2.1.2.2)	Patient‐specific 3D‐printed (section 2.1.2.2.1)
			Non‐patient specific scaffolds^†^ (section 2.1.2.2.2)
	Bone transport osteogenesis (section 2.1.3)		
	Heterotopic autografts (section 2.1.4)	Non‐vascular autograft (section 2.1.4.1)	
		Vascular autografts (section 2.1.4.2)	Microvascular transfer (section 2.1.4.2.1)
			Ulnar rollover transposition (section 2.1.4.2.2)
	Manus lateral translation (section 2.1.5)		
	Orthotopic autografts (section 2.1.6)	Ex situ treatment of the tumor (section 2.1.6.1)	Intraoperative radiation therapy (section 2.1.6.1.1)
			Pasteurization (section 2.1.6.1.2)
			Cryotherapy (section 2.1.6.1.3
		In situ treatment of the tumor (section 2.1.6.2)	Microwave ablation with cementoplasty (section 2.1.6.2.1)
			Cementoplasty alone (section 2.3.5)
Limb not reconstructed (section 2.2)	Scapular tumors	Partial or complete Scapulectomy (section 2.2.1)	
	Ulnar tumors	Partial ulnectomy (section 2.2.2)	
	Radial tumors*	Limb shortening (section 2.2.3)	

Abbreviations: 3D, three‐dimensional; ITAP, intraosseous transcutaneous amputation prosthesis.

^†^ Single case report. The case report did not demonstrate successful growth of new bone in the critical size defect.

* The limb was not reconstructed from the perspective that the critical bone defect was not reconstructed. However, the limb was stabilized with a plate to allow weightbearing.

**TABLE 2:** Surgical limb sparing techniques reported for clinical cases for the most common anatomic sites of osteosarcoma in dogs.

Anatomic site	Surgical techniques
Proximal humerus	Cortical allograft Orthotopic autograft (irradiated)
Distal radius	Cortical allograft Metal (off‐the‐shelf or patient‐specific [3D printed]) endoprosthesis implants Bioreplaceable endoprosthesis Bone transport osteogenesis Heterotopic autograft Manus translation Orthotopic autograft (pasteurized, irradiated, cryotherapy, microwave ablation)
Proximal femur	Cortical allograft Metal or patient‐specifc (3D printed) endoprosthesis implants
Distal femur	Cortical allograft Orthotopic autograft (irradiated, cryotherapy)
Proximal tibia	Cortical allograft Metal or patient‐specifc (3D printed) endoprosthesis implants Bone transport osteogenesis Orthotopic autograft (cryotherapy)
Distal tibia	Cortical allograft Metal or patient‐specifc (3D printed) endoprosthesis implants Bone transport osteogenesis Orthotopic autograft (irradiated)

Abbreviation: 3D, three dimensional.

Techniques broadly fall into two categories: (1) reconstruction of the critical bone defect and (2) no reconstruction needed. Currently, the majority of techniques involve reconstruction. The purpose of this review is to describe the existing limb sparing techniques for dogs, discuss the benefits and complications of each technique, and guide clinical decision making for those interested in performing limb‐sparing. The review first organizes techniques via whether reconstruction is needed or not and subsequently has techniques organized by anatomical site they have been applied in.

### 1.1 General indications and contraindications

Historically, limb‐sparing was developed for dogs that were anticipated to not tolerate a limb amputation clinically, wherein the function and quality of life of the dog would be significantly impacted and considered unacceptable post‐amputation. Recognizing poor candidacy for an amputation is a subjective assessment, and it remains an indication for limb sparing. Another indication is in cases where the owners are unwilling to amputate their pet's limb, which can be for a number or reasons, including religious and cultural. However, limb sparing is one of the now many treatment options for the local disease for appendicular osteosarcoma. As such, limb sparing can be offered with all other treatment options, including curative intent (sometimes referred to as definitive options) and palliative, that are appropriate for each case and does not to be reserved for dogs that are poor candidates for amputation.

The main medical contraindication in cases with neoplasia is a dog with detectable metastatic disease. Historically, a primary tumor that affected >50% of the length of the bone was deemed a contraindication; however, this criterion is often challenged and not adhered to. Tumors that involve the entirety of the soft tissues surrounding it (360° coverage) and tumors with marked edema instead of a firm/definable soft tissue component are considered contraindications.^10^ Whether a pathologic fracture is a contraindication is controversial. The rationale for a pathologic fracture being a contraindication is that the pseudocapsule of the tumor would be disrupted, allowing the release of tumor cells in the surrounding tissues thereby increasing the risk of local recurrence. In one study, pathologic fracture was not a negative prognostic factor for local recurrence but this was based on retrospective data and ultimately few cases.^11^ One proposed approach can be that a non‐displaced or minimally displaced pathologic fracture is not a contraindication to limb sparing (where the pseudocapsule remained intact) whereas a displaced pathologic fracture is (where the pseudocapsule is disrupted). Undoubtedly, indications and contraindications may change due to the mostly subjective nature of these assessments as described and based on the development of new implants and new techniques.

## 2 LIMB‐SPARING TECHNIQUES: BRIEF DESCRIPTIONS

### 2.1 Limb‐sparing techniques with limb reconstruction

The initial step for all techniques that lead to reconstruction of the limb is to remove the affected segment of bone. To facilitate this, the soft tissues circumferentially around the affected segment of bone must be dissected away. In cases of neoplasia, it is imperative that the tumor pseudocapsule is not penetrated during the soft tissue dissection. Being that most tumors involve the metaphysis of the bone, an osteotomy is made in the diaphysis of the bone 3 to 5 cm away from the closest margin of the tumor. The joint capsule adjacent to the tumor is incised circumferentially, allowing the bone segment to be excised. This creates a critical‐sized defect. The next step is to reconstruct the bone defect.

#### 2.1.1 Cortical allografts

**FIGURE 1:**
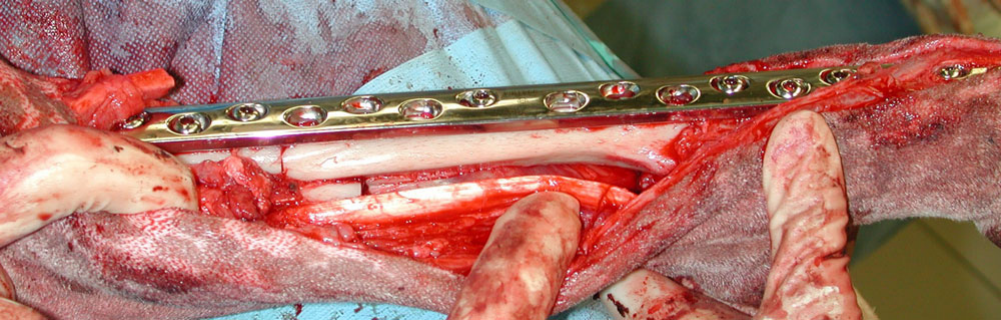
Intraoperative image of a cortical allograft placed in the radial defect after tumor excision. The elbow is on the left side of the image. Photograph courtesy: Dr Peter Walsh.

**FIGURE 2:**
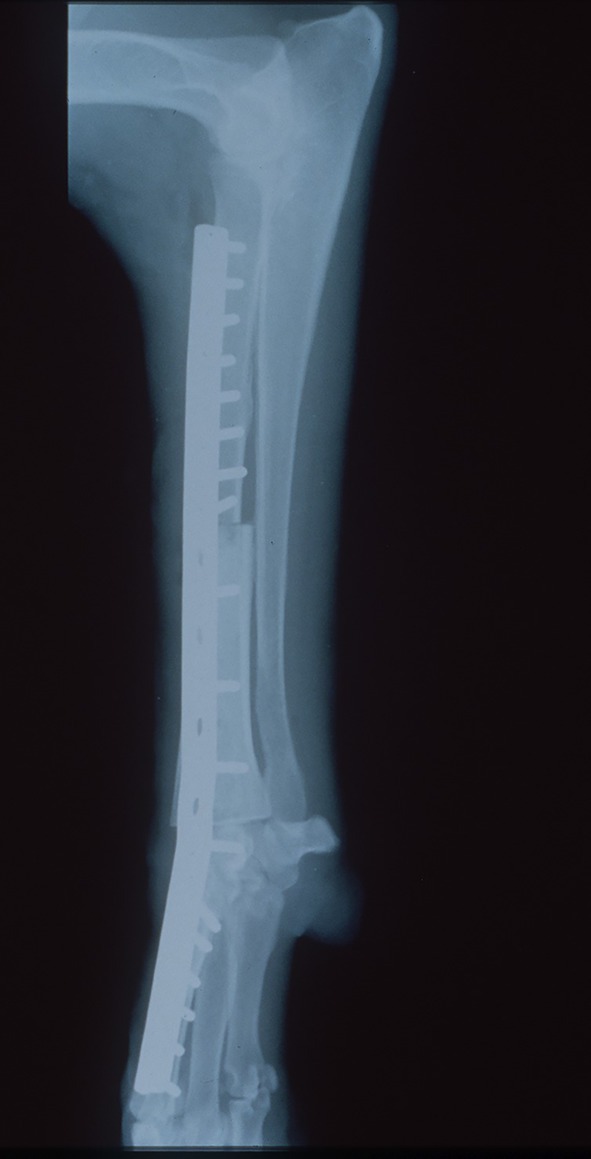
Lateral radiograph of a limb‐spare following reconstruction with a cortical allograft. Photograph courtesy: Dr Peter Walsh.

Cortical allografts are whole bones harvested from a deceased donor dog to fill in the critical‐sized defect.^12^ The donor bones are kept frozen in a bone bank. A specimen that matches the size of the recipient as close as possible is thawed and then used to repair the defect. It is cut to the appropriate length and the medullary cavity of the allograft is reamed and polymethylmethacrylate (PMMA) is inserted into it. The allograft is inserted into the critical‐sized defect, and secured with a plate and screws (Figures 1 and 2). Healing of cortical allografts occurs via the patient's vasculature penetrating the allograft and the body mounting an immunologic response that demineralizes and resorbs the implanted allogenic bone while simultaneously substituting it with host bone.^13^ This is a slow process called creeping substitution which can take months to years to occur and has poor strength overall.^13^, ^14^, ^15^

#### 2.1.2 Endoprosthetic implants

##### Metal endoprostheses

###### Off‐the‐shelf endoprostheses

**FIGURE 3:**
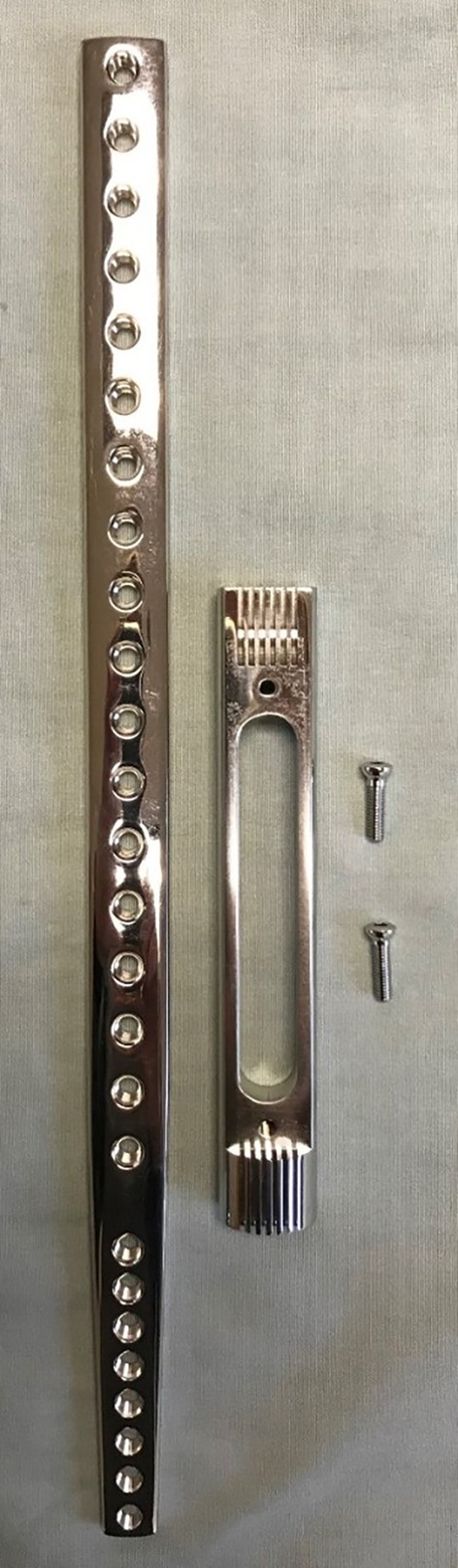
Commercially available metal endoprosthesis with the purpose‐made screws required to connect the limb sparing plate to the endoprosthesis. Photograph courtesy: Dr Bernard Seguin.

**FIGURE 4:**
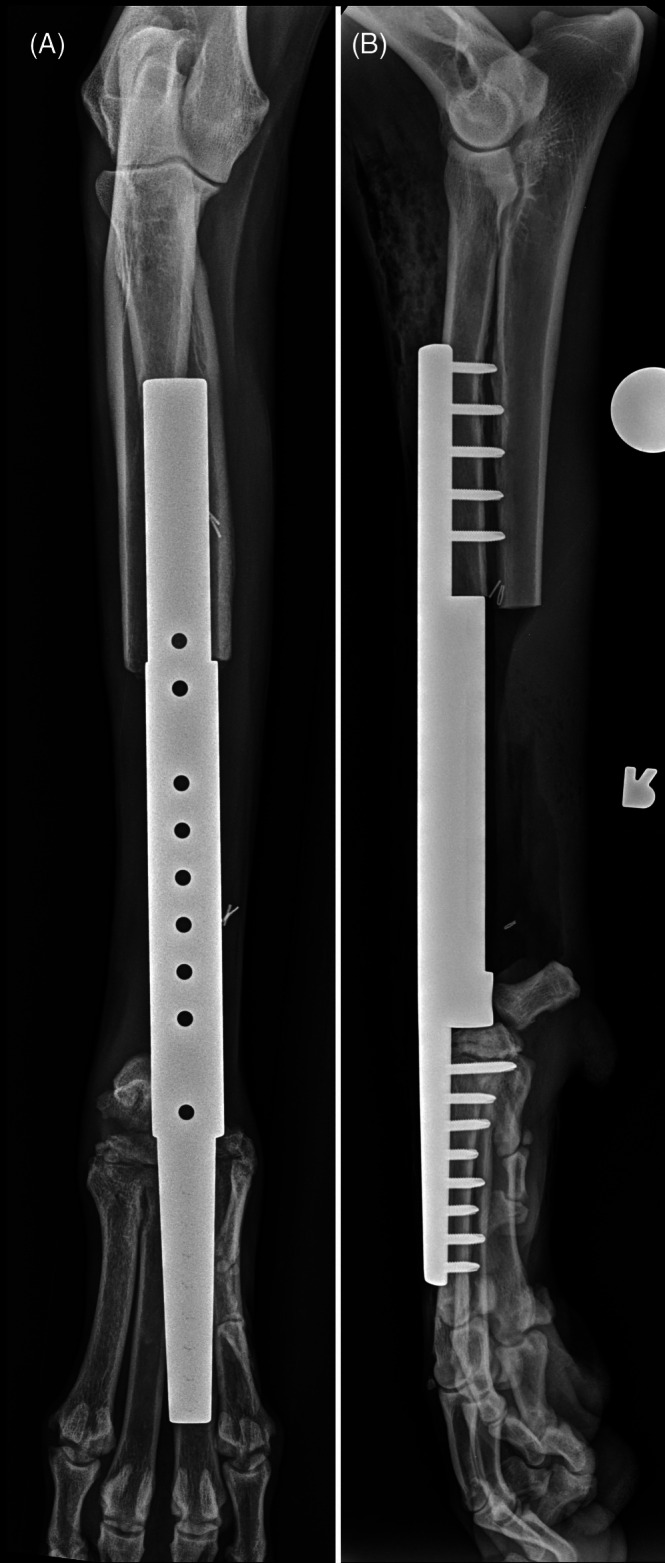
(A) Craniocaudal and (B) lateral radiographs of a limb‐spare following reconstruction with a commercially available metal endoprosthesis. Photograph courtesy: Dr Bernard Seguin.

The earliest form of limb‐sparing with metal eoprostheses in dogs utilized off‐the‐shelf metal implants. Currently available metal endoprostheses are made with stainless steel and are either 98 mm or 122 mm long (Movora, https://movora.com, VOI radius plate, Veterinary Orthopedic Implants, Burlington, Vermont). These endoprostheses require purpose‐made screws, also from Movora, to be affixed to the plate (Figures 3 and 4). With this technique, the metal endoprosthesis is inserted into the critical‐sized defect instead of an allograft and the endoprosthesis is secured in place with a plate in screws similar to the allograft technique.

###### Patient‐specifc endoprostheses

**FIGURE 5:**
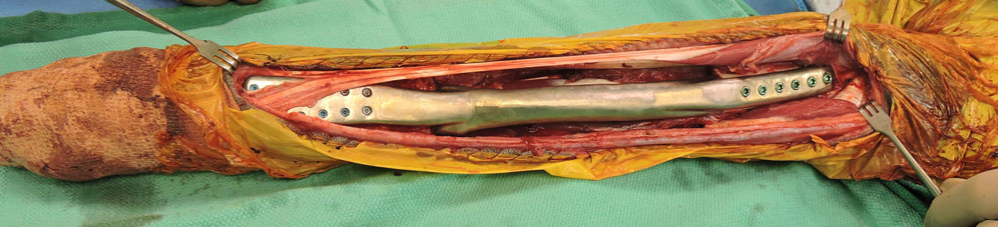
Intraoperative picture of a three‐dimensional (3D)‐printed personalized metal endoprosthesis placed in the radial defect after tumor excision. The elbow is on the right side of the image. Photograph courtesy: Dr Bernard Seguin.

**FIGURE 6:**
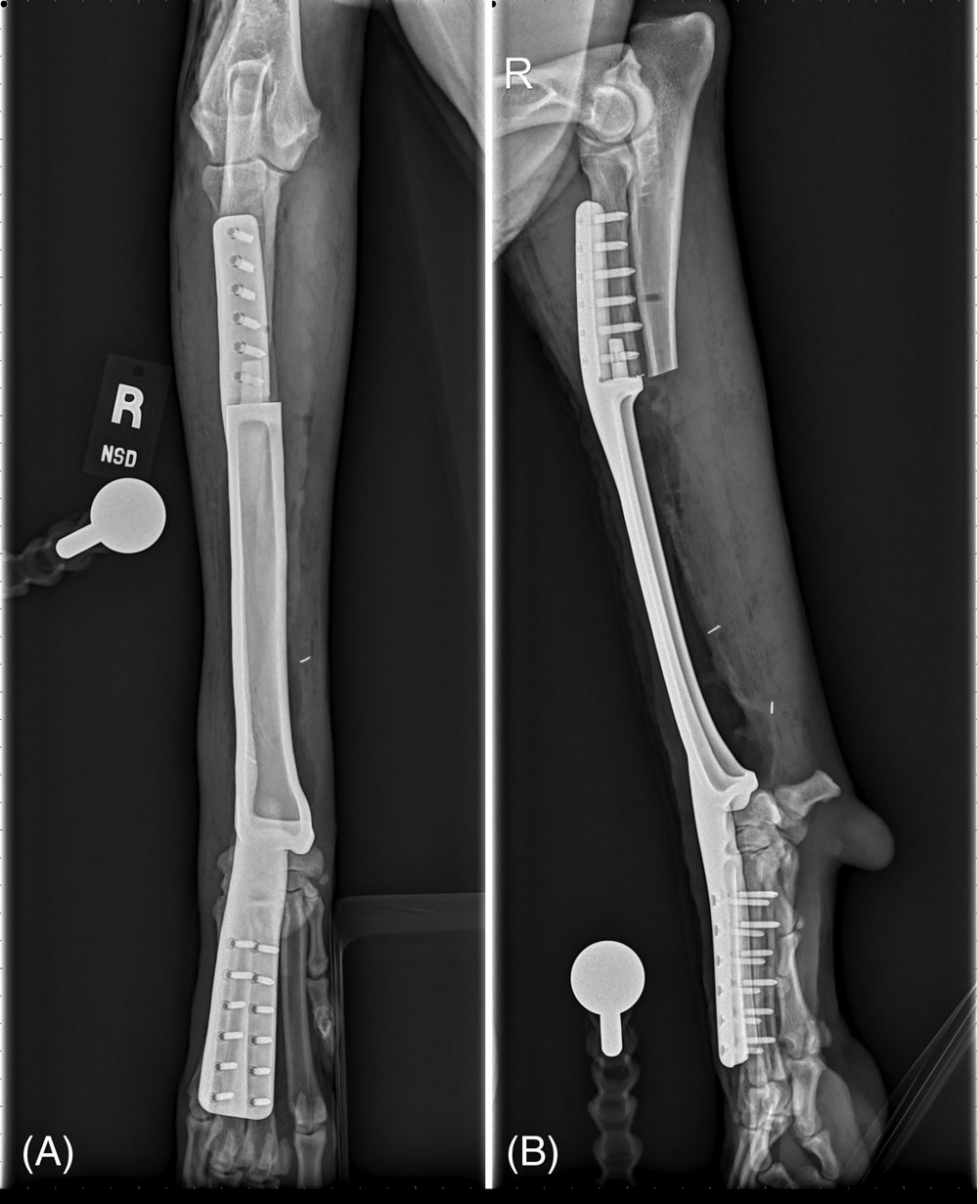
(A) Craniocaudal and (B) lateral radiographs of a limb spare following reconstruction with a three‐dimensional (3D)‐printed personalized metal endoprosthesis. Photograph courtesy: Dr Bernard Seguin.

The advancement and increased availability of three‐dimensional (3D)‐printing technology and advanced imaging (computed tomography [CT]) have allowed for the design and production of patient‐specific implants in silico that can then be 3D‐printed and placed into the critical‐sized defect. These implants are typically made from titanium (Figures 5 and 6).^16^, ^17^

##### Bioreplaceable endoprostheses

Bioreplaceable endoprostheses in dogs can similarly be patient‐specific^18^, ^19^ or not.^20^ The process overall for the patient‐specifc bioreplaceable implants is the same as with the patient‐specific 3D‐printed metal endoprostheses; however, the material is biodegradable and bioreplaceable meant to act as a scaffold to allow bone ingrowth and be replaced by viable healthy bone.^18^, ^19^ A common material reported thus far is β‐tricalcium phosphate with or without polycaprolactone.^18^, ^19^

For the non‐patient specifc approach, there has only been a single case of this in which a segment of the distal radius was removed and the limb was stabilized with a pancarpal arthrodesis and two plates placed orthogonally.^20^ Cancellous bone autograft and cancellous allograft bone chips with a non‐immunogenic copolymer scaffold were inserted at the ostectomy site to fill the defect created.^20^

#### 2.1.3 Bone transport osteogenesis

Bone transport osteogenesis (BTO), also termed distraction osteogenesis, has been described as a strategy to perform veterinary surgical limb‐sparing, using slow and gradual distraction of the healthy bone segment, at either end of a defect to fill it with new bone.^21^, ^22^, ^23^, ^24^, ^25^, ^26^, ^27^ An external skeletal fixator (ESF), which can be bilateral or circular rings, is used to facilitate the distraction.^21^, ^22^, ^23^, ^24^, ^25^ With BTO, implants can be removed and infection is not reported with this technique.^22^, ^24^ However, owners are responsible for distracting the bone segment every day and the time post‐surgery until ESF removal has been reported to take up to 350 days.^24^ The majority of published BTO cases are longitudinal, termed as such due to the distraction occurring longitudinally, from proximal to distal. With the standard distraction rate of 1 mm per day, distracting longitudinally is a very long process.^22^, ^23^, ^24^ In an attempt to complete distraction in less time, the transverse BTO approach was developed and involves distraction in the transverse plane relative to the dominant bone axis, that is, from lateral to medial.^25^ In one case series, osteotomies of the ulna were performed to create a segment of ulna the length of the radial defect to be displaced from lateral to medial.^25^

#### 2.1.4 Heterotopic autografts

##### Heterotopic non‐vascularized autografts

Heterotopic autografting is the transplantation of a patient's own tissue into a different location within the body. A non‐vascularized ulna autograft has been reported in a single case report.^28^ The proximal ulna (but distal to the elbow joint) was harvested without preserving its blood supply. The ulna was then placed in the critical‐sized defect created from the tumor excision and maintained in place with plates and screws.^28^

##### Heterotopic vascularized autografts

###### Microvascular transfer

In the context of surgical limb‐sparing, autografting—particularly with vascularized autografts—may provide advantages over the use of cortical allografts such as increased osteocyte survival, less postoperative infections, improved incorporation of graft into host bone, and lower chances of a non‐union healing.^29^, ^30^, ^31^, ^32^ Vascularized bone autografts have been shown to have faster healing and more bone and callus formation than non‐vascularized autografts.^33^, ^34^ However, heterotopic vascularized autografts may require microvascular surgery, which requires special equipment such as an operating microscope and training in microvascular surgery techniques for the surgeon. The most commonly used bone for microvascular transfer of a heterotopic graft is the distal ulna. The distal ulna is harvested by preserving its periosteal blood supply and the artery and vein (caudal interosseous) that supply and drain it. The vessels are transected to allow their transfer into the critical‐seize defect and the vessels are anastomosed to local vessels in the recipient site. The ulnar graft is then secured in this anatomic site with a plate and screws.

###### Ulnar transposition

**FIGURE 7:**
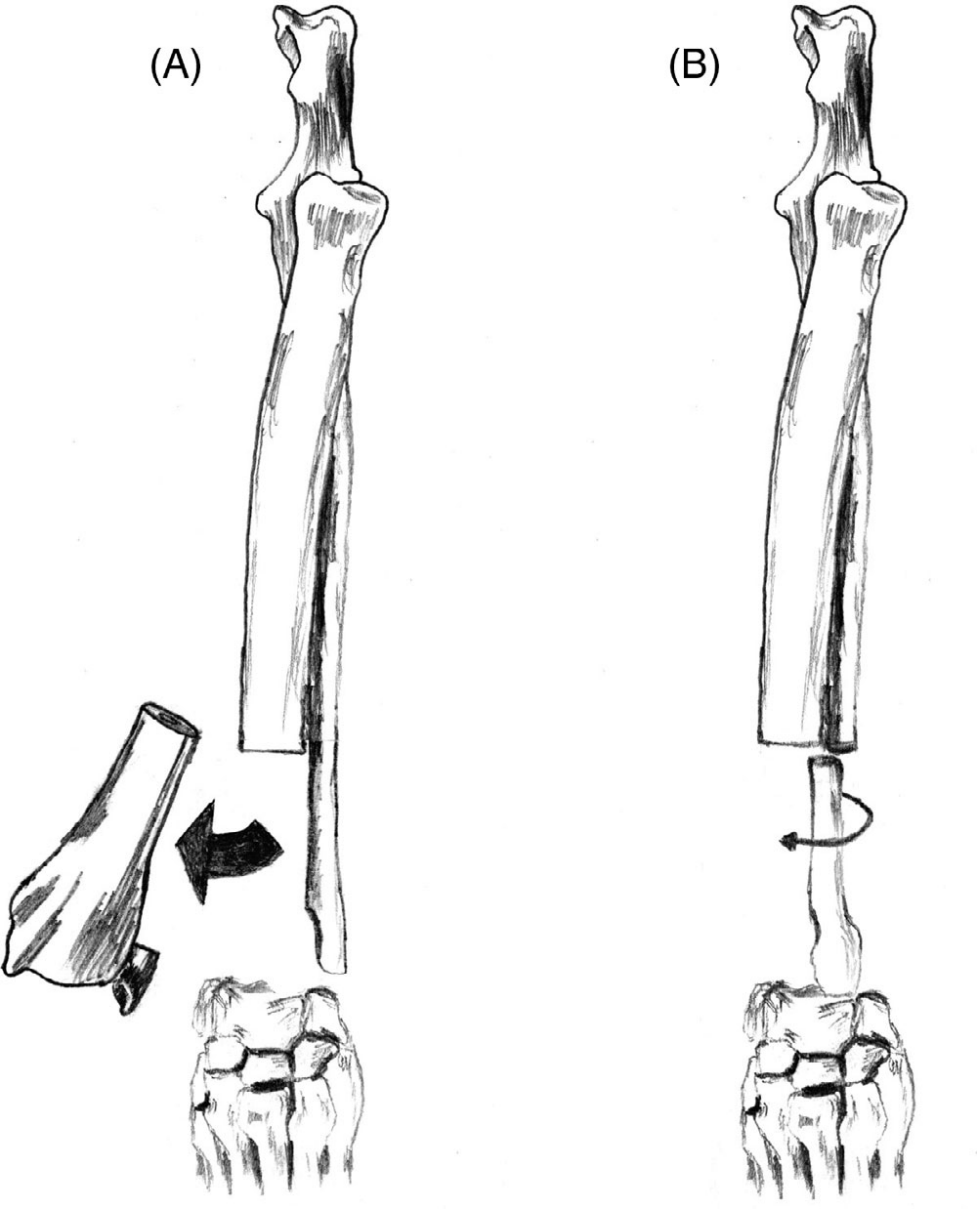
Illustration of the ulnar rollover transposition limb‐sparing technique. (A) The distal radius is excised, as with most other techniques for the distal radial site. (B) The distal ulnar graft is created and pivoted around the blood vessels caudal interosseous artery and vein (not shown), which support the viability of the graft, to rest in the distal radial defect. Reprinted with permission.^83^

**FIGURE 8:**
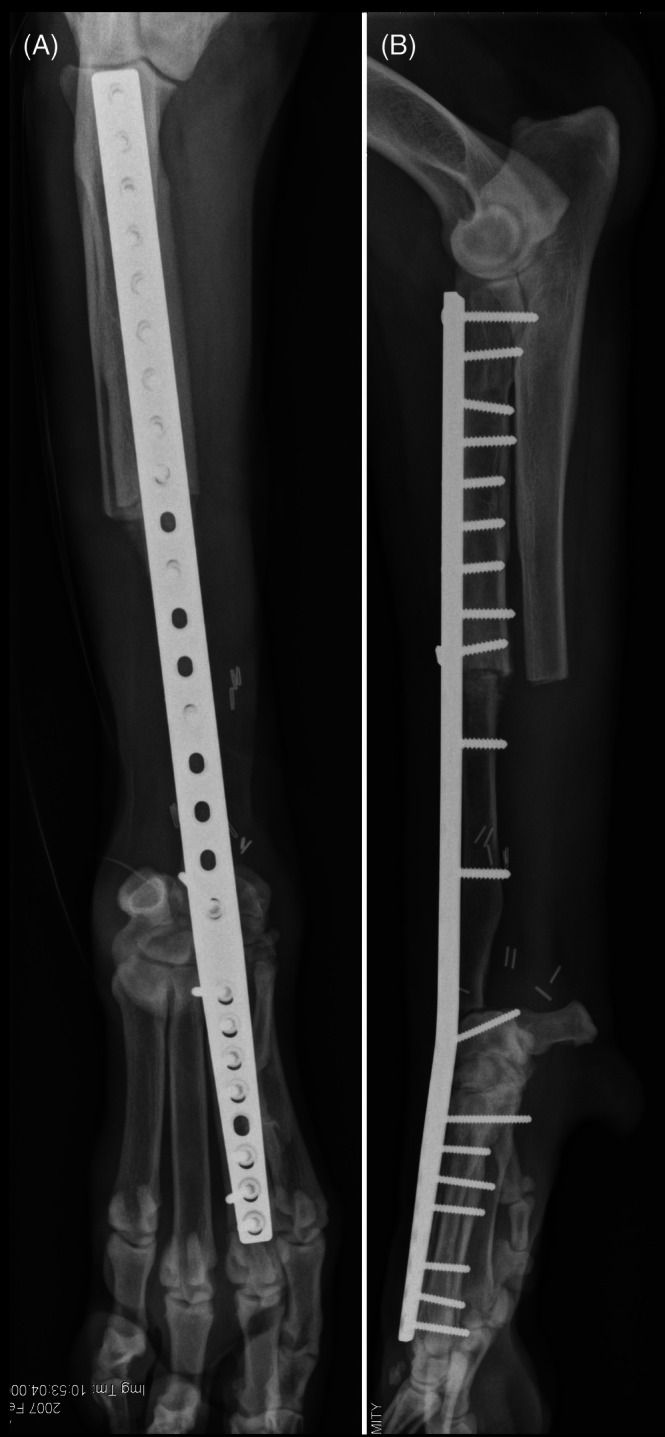
(A) Craniocaudal and (B) lateral radiographs of a limb spare following reconstruction with the ulnar rollover transposition technique. Photograph courtesy: Dr Bernard Seguin.

Ulnar transposition (UT), or ulna rollover transposition, is a surgical technique that has been used specifically to conduct surgical limb‐sparing at the distal radius (Figures 7 and 8). With this technique, the caudal interosseous artery and vein are kept intact and the ulna graft is pivoted around these vessels to then be positioned in to the radial defect, negating the necessity for microvascular surgery.^35^

#### 2.1.5 Lateral manus translation (LMT)

**FIGURE 9:**
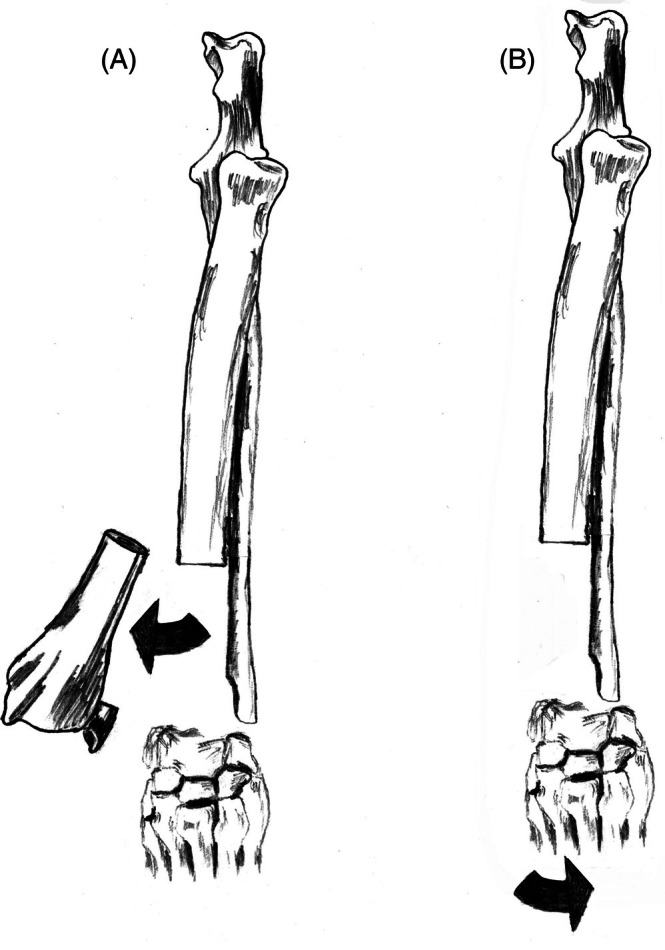
Illustration of the manus lateral translation limb sparing technique. (A) The distal radius is excised, as with most other techniques for the distal radial site. (B) The manus is moved laterally such that it is distal to and in contact with the distal end of the ulna. Reprinted with permission.^36^

**FIGURE 10:**
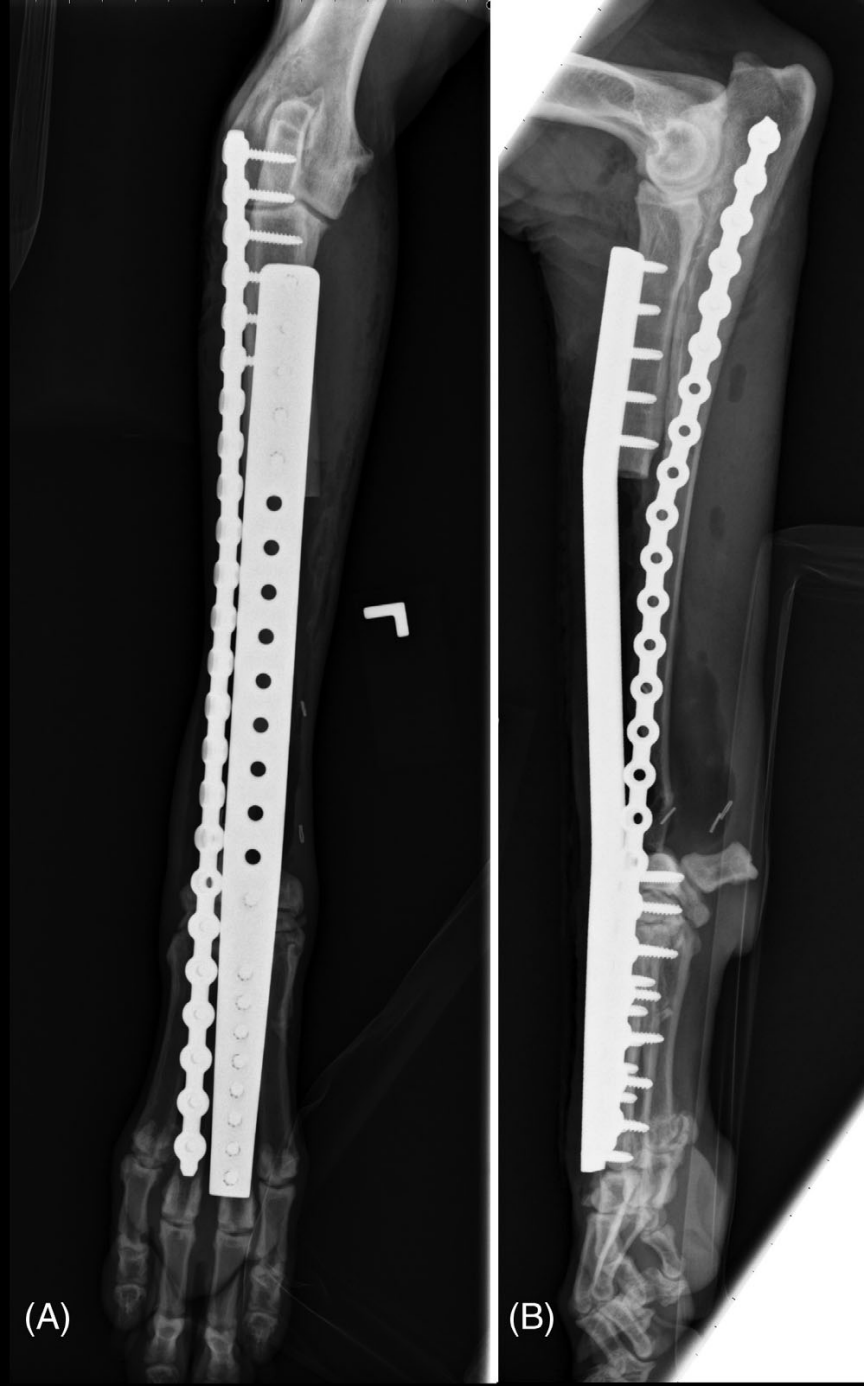
(A) Craniocaudal and (B) lateral radiographs of a limb spare following reconstruction with the lateral manus translation technique. Photograph courtesy: Dr Bernard Seguin.

With this technique, the ulna remains intact (except for the ulnar styloid process being removed). A lateral translation of the manus such that the ulna is in contact with and can be secured to the intermedioradial bone with bone a plate is performed (Figures 9 and 10).^36^

#### 2.1.6 Orthotopic autografts

##### Ex situ treatment of the tumor

###### Irradiated autografts

Another strategy that can be used in surgical limb‐sparing if a tumoral autograft is to be used is treating the harvested bone with the tumor with a single fraction, high‐dose radiation therapy intraoperatively (50–300 Gy) prior to placement back into the patient.^37^, ^38^ With this approach, irradiation of the autograft occurs extracorporeally. In a report of 13 dogs receiving irradiated autografts at various anatomic sites, the radius and one of the tibia OSA bones were irradiated ex vivo after harvesting the autograft whereas the remaining bones were irradiated intraoperatively extracorporeally: an osteotomy is made in the diaphysis and while the segment with the tumor is still “attached” at the joint, the segment is exteriorized of the body by “flipping” the segment and suspend the segment to be irradiated with a horizontal beam.^37^ Once the radiation therapy is completed, the segment is placed into its orthotopic position and stabilization with a plate, interlocking nail, intramedullary pin, alone or in combination.^37^

###### Pasteurized autografts

Pasteurized tumoral autografting is a technique in which the segment of bone affected by neoplasia can be excised, pasteurized to kill the tumor cells, and then implanted back as an autograft.^39^, ^40^ The pasteurization process involves heating the segment of bone at 65°C for 40 min.^39^, ^40^

###### Frozen autografts (cryotherapy)

Another method of preparing orthotopic autografts that has been employed clinically is freezing the autograft to −196°C for 20 min, which can be achieved with liquid nitrogen, as a method to kill tumor cells.^41^, ^42^, ^43^

##### In situ treatment of the tumor—microwave ablation with cementoplasty

A 2023 single case report described microwave ablation (MWA) and cementoplasty as a minimally invasive surgical approach to limb‐sparing at the distal radius due to OSA.^44^ In this approach, both the MWA and cementoplasty is believed to play a role in bone tumor control and the cementoplasty provides additional palliation and mechanical support for the affected limb.^44^ The cementoplasty was accomplished using a synthetic calcium‐phosphate bone substitute (BIOCERA‐VET‐OSA, Theravet, Belgium).^44^

### 2.2 Limb‐sparing techniques without limb reconstruction

#### 2.2.1 Scapulectomy

Limb‐sparing via scapulectomy can be considered for treatment of scapular tumors.^45^, ^46^, ^47^, ^48^, ^49^ Scapulectomy can be partial or total based on the location and extent of the pathology.

#### 2.2.2 Partial ulnectomy

Partial ulnectomy has been described for surgical removal of tumors affecting the distal ulna wherein the affected portion of ulna is removed while maintaining the elbow joint.^50^, ^51^, ^52^, ^53^ When the excision of the distal ulna requires excising the styloid process, reconstruction of a lateral collateral ligament is not required.^52^, ^53^

#### 2.2.3 Limb shortening

In a method that avoids the need to fill the defect created by distal radius ostectomy and with the aim to avoid the high complication rate of endoprosthetic limb‐sparing, a limb‐shortening procedure in a dog with distal radius OSA was reported.^54^ After excision of the distal radius, the manus was placed directly against the osteotomized radius thus closing the gap created intraoperatively.^54^

### 2.3 Alternative limb‐sparing techniques

#### 2.3.1 Surgical fixation of pathologic fractures and transtumoral plating

Surgical fixation of pathologic fractures has been reported in one study of 16 dogs wherein the tumor was not excised.^55^ No complications were reported regarding the fixation methods. Despite lack of follow‐up information on many of the included fractures, the authors concluded that fracture fixation is feasible for dogs with OSA as a palliative measure.^55^

Transtumoral plating was reported in 1 dog with distal radial OSA for palliative reasons where pathologic fracture was suspected.^56^ A plate was contoured to the mass and applied to the cranial surface of the radius and carpal and metacarpal bones without excision of the bone tumor.^56^ Limb use was good in that case until 130 days postoperatively due to local disease progression.^56^

#### 2.3.2 Radiation therapy with surgical stabilization

Stereotactic body radiation therapy (SBRT) and stereotactic radiosurgery are forms of radiation therapy where high doses of radiation are delivered over either a few doses or as one dose respectively.^57^, ^58^, ^59^ Both have been used in cases of appendicular OSA in dogs as a non‐surgical limb‐sparing technique.^57^, ^58^, ^59^ Surgery has been performed post‐SBRT to either address a pathologic fracture or as a prophylaxis to prevent a pathologic fracture.^60^, ^61^ Complication rate is very high when surgery has been performed after SBRT with the majority of complications being considered major.^60^, ^61^ Consequently, surgical stabilization following SBRT is not recommended.^60^, ^61^

Similarly, surgical stabilization paired with non‐stereotactic RT has been described.^62^ In contrast to the cases previously mentioned that received stereotactic body radiation therapy and surgical stabilization, dogs treated with non‐stereotactic radiation and surgery appeared to have minimal major complications.^62^

#### 2.3.3 Intraosseous transcutaneous amputation prosthesis (ITAP)

ITAP in dogs is when a partial limb amputation is performed and then followed up with implantation of an endoprosthesis into the remaining bone segment (usually the radius or tibia) which has an external component (outside the skin) that connects to an exoprosthesis for weightbearing and transfer of ground reaction forces.^8^, ^9^

#### 2.3.4 Histotripsy

Histotripsy is a non‐thermal, non‐invasive, and non‐ionizing focused ultrasound ablation technique that uses highly controlled, high‐amplitude ultrasound pulses to generate acoustic cavitation bubble clouds and mechanically disintegrate tissue. Histotripsy has been used as an alternative limb sparing approach to eradicate osteosarcoma in dogs in vivo. However, at the time of writing, the technique is still considered investigational. All of the dogs reported in the published literature have had an amputation within 18 to 24 h post‐treatment and therefore the long‐term outcome is unknown.^63^, ^64^

#### 2.3.5 Cementoplasty alone

The use of calcium phosphate bone cement has been trialed as a limb‐sparing technique in a study of 12 dogs where the cement was injected directly into the tumor to study the feasibility and utility of this approach for palliation and prevention of pathologic fracture.^65^

## 3 CONSIDERATIONS FOR LIMB‐SPARING BY ANATOMIC SITE

### 3.1 Radius

The distal radius is one of the two most common anatomic sites for osteosarcoma in dogs and is the anatomic site with the most cases and data reported for limb sparing. Because osteosarcoma most commonly affects the metaphysis, arthrodesis of the adjacent joint is a common consequence of many limb sparing techniques. Pancarpal arthrodesis leads to the best functional outcomes given the minimal loss of function compared to arthrodesis of the stifle, hock, or shoulder.^66^

Cortical allografts have been described to treat primary bone tumors,^66^, ^67^, ^68^, ^69^, ^70^, ^71^, ^72^, ^73^, ^74^ and less frequently to repair comminuted fractures of the radius.^75^

Infection is the most common complication with almost every technique for limb sparing of the distal radius, with the exception of BTO. Infection has been shown to decrease local recurrence and increase survival time.^70^ One study of 47 dogs found that dogs with distal radius OSA treated with cortical allograft and chemotherapy lived longer if they developed an infection. Dogs with a surgical site infection were half as likely to have metastatic disease.^74^ The median survival for dogs without an infection was 228 days and 480 days for those with an infection.^74^ The association between an infected limb spare and longer survival has been reported in additional studies.^11^, ^68^, ^76^, ^77^

In one study of 42 dogs, allograft and screw failures were increased in non‐cemented allografts with both cemented and non‐cemented allografts having equally high infection rates of 70%.^71^ Cemented allografts have shown delayed healing but the mechanical advantage of the cement outweighs the delayed healing.^71^

One study of 142 dogs reported the rate of amputation following limb‐sparing of the distal radius was 16% due to non‐treatable complications.^76^ Although the study included different surgical techniques for the limb sparing, the majority (76%) were cortical allografts.^76^ The other techniques included were UT and metal endoprosthetics.^76^

A study of 45 dogs with distal radius OSA studied two generations of pre‐made endoprostheses (Veterinary Orthopedic Implants, now Movora, https://movora.com/).^11^ No difference in complication frequency between either generation was identified, and a positive relationship between complication occurrence and survival time was observed.^11^ The overall complication rate was high at 96%. A total of 31% were minor and 64% were major. In this study, minor complications comprised mild implant‐related complications or mild/moderate surgical infections that were treated conservatively and major complications comprised local recurrence or complications that required amputation or revision surgery.^11^ Infection developed in 78% of cases, implant‐related complications in 36%, and local recurrence in 24%.^11^ Notably, local recurrence was not associated with complete versus incomplete excision of the tumor and was significantly lower when an infection developed.^11^ Amputation due to major complications occurred in 20% of cases.^11^

Several single‐case reports have described endoprosthetic limb‐sparing on the canine radius. One report described the use of a custom‐made tantalum endoprosthetic implant to fill the defect after OSA excision.^78^ Limb function was good and it was suggested that use of the custom tantalum implant was easier than use of more common limb‐sparing implants.

Another report involved a dog with a large distal radial OSA where excision of the carpal bones was performed en bloc with the distal radius and ulna.^79^ An off‐the‐shelf metal endoprosthesis was used, making contact distally with the metacarpal bones.^79^ This dog experienced a postoperative infection managed with antibiotics and, subjectively, regained normal ambulation.^79^

Outcomes of limb‐sparing of the distal radius with patient‐specific endoprostheses made using additive manufacturing (3D‐printing) have been reported in three studies.^16^, ^17^, ^80^ Postoperative complications reported included local recurrence, implant problems, and infection.^16^, ^17^, ^80^ The biggest advantage of this approach is the customizability of the implant that additive manufacturing allows.^17^ A major disadvantage of this approach is the time needed to design and manufacture the implant—time during which the tumor can continue to grow. To mitigate this, neoadjuvant intra‐arterial carboplatin was used in one study, and tumor growth between workup and surgery was not observed.^17^

One case of a radial diaphyseal non‐union was treated with a patient‐specific β‐tricalcium phosphate endoprosthesis where the region of the non‐union was excised and the defect was filled with the implant.^18^ Complete healing of the radius was documented at 4 months postoperatively and the plate was removed 10 months afterwards. In another case, a patient‐specific endoprosthesis made from polycaprolactone and β‐tricalcium phosphate was used for limb‐sparing of the distal radius with OSA.^19^ Local recurrence occurred 4 months postoperatively and the dog died 190 days after the diagnosis. No definitive tests were performed to see if bone ingrowth had occured.^19^

The use of a non‐specific, bioreplaceable endoprosthesis has been reported in a dog with distal radius OSA.^20^ The dog had normal limb function 14 months postoperatively.^20^

Longitudinal BTO has been reported most commonly for dogs with distal radial osteosarcoma as seen in a study of nine dogs.^24^ Wire failure (categorized as a minor complication) was most common complication. Postoperative limb function was rated good to excellent in the majority of dogs.^24^

Transverse BTO has been applied to limb spare a distal radial site using a segment of ulna in a single case report.^25^ Although complications occurred, distraction across the 8.4 cm longitudinal defect in this dog was achieved in only 23 days—a much shorter time frame than with longitudinal BTO.^25^ The dog experienced a fracture at the distal docking site after removal of the ESF, which eventually required stabilization with a plate.^25^

In an ex vivo mechanical study, cortical allografted limbs were biochemically superior and resisted greater amounts of bending than UT limbs; however, both sets of limbs withstood a yield load that is greater than triple the expected peak vertical ground forces of a dog at a trot.^81^

The outcomes of dogs with distal radius OSA treated with UT have been reported.^82^, ^83^ Complications include infection, biomechanical problems (implant loosening, implant failure, fracture of the proximal radius), and local recurrence.^82^, ^83^ In one study of 27 limbs, using nuclear scintigraphy, histology, or both, viability of the UT grafts was assessed as viable in 17 limbs, non‐viable in three and unknown in seven.^83^

The case of ulnar heterotopic non‐vascularized autograft had a survival time of 282 days; however, the dog experienced a construct failure (no surgical correction required) and local recurrence was suspected.^28^

In a study of 18 dogs that underwent LMT surgery, three had fractures of the lateral distal cortex of the ulna during the osteotomy, 10 developed postoperative infections (1 amputation due to severe infection), four had local recurrence, and biomechanical failure in six dogs due to screw or plate loosening or breakage.^36^ With limb function subjectively being assessed as acceptable, authors concluded that the LMT procedure was easier to perform than UT.^36^

The study by Liptak et al. included one case of radial mid‐diaphyseal OSA which underwent limb‐sparing with an irradiated tumoral autograft.^37^ No complications occurred from that case and the dog was alive at the time of publication with a survival time of greater than 500 days.^37^ Another study focused on irradiation of the distal radius in five dogs which was completed extracorporeally, as previously described (see section 2.1.6.1.1).^38^ In this instance, pancarpal arthrodesis was not performed and the segment was stabilized with a plate.^38^ All dogs had a complication: three dogs were treated with amputation and two were treated with pancarpal arthrodesis.^38^ This approach to preserve function of the carpal joints is not recommended.^38^

A single case report described microwave ablation (MWA) and cementoplasty as a minimally invasive surgical approach to limb‐sparing at the distal radius due to OSA.^44^ Three months postoperatively, the dog developed a pathologic fracture of the radius and was managed conservatively with bandaging. The dog developed osteomyelitis at the site and required antibiotic therapy at 7 months postoperatively. The dog died due to metastatic disease progression 541 days after surgery.^44^

Pasteurized tumoral autografts was first reported in a single case report.^39^ No complications were reported and the dog had fair to good limb function with no evidence of disease 56 months postoperatively.^39^ That case report was followed by a larger study of 13 dogs with distal radius OSA.^40^ Complications were local recurrence in two dogs, autograft infection in four dogs and implant failure in three dogs due to screw loosening^40^ Out of 13 dogs, 12 ended up with good limb function postoperatively (subjective assessment) and one had fair limb function.^40^

Frozen radial autograft has been described in one dog with radial OSA.^43^ The dog was alive and had subjectively good limb function 13 months postoperatively and had no evidence of metastatic disease but subsequently developed hemoperitoneum with evidence of splenic and hepatic masses which the dog succumbed from.^43^

Distal radius ostectomy and limb shortening was performed in one dog where 6 cm of radius was excised in a 24 kg Labrador Retriever.^54^ Limb function was deemed adequate by authors; however, there were three postoperative complications warranting treatment: infection requiring antibiotics long‐term, focal skin necorsis requiring surgical revision, fracture of third metacarpal through a screw hole requiring surgery and external prosthesis.^54^

ITAP using customized 3D‐printed implants has been reported in three clinical cases of distal radius OSA. No local recurrence was reported and the dogs had subjectively good use of the limb. Fracture of the ITAP occurred in one case requiring replacement which was successful. Two dogs were euthanized due to metastatic disease at 12 and 17 months postoperatively with the third being alive at the time of publication 18 months postoperatively.

### 3.2 Ulna

Ulnar cortical allografts have been reported in dogs with comminuted fractures of the ulna^75^, ^84^ and dogs with ulnar OSA.^66^, ^67^, ^68^, ^70^, ^72^, ^73^ In a study of intercalary allografts in 17 dogs, every implant failure occurred in the ulna.^73^ The intercalary graft was to reconstruct the diaphysis and joint function was preserved.^73^ Implant failure was associated with the use of non‐cemented allografts.^73^ That report also highlighted significant associations between formation of non‐unions and use of PMMA cement as well as use of a biodegradable matrix that delivered cisplatin locally.^73^ Intercalary resection and reconstruction is considered inappropriate for tumors of the ulna based on the high risk of implant failure^73^ and because reconstruction is unnecessary.^51^, ^52^, ^53^ Instead, ulnectomy without reconstruction is most commonly used where feasible.

A 1991 study reporting on ulnar ostectomy found that this procedure was associated with a high local recurrence rate in 5/8 dogs.^51^ A 2013 study reported 11 dogs with ulnar OSA that were treated with ulnar ostectomy: five had incomplete resection of the tumor and one of these dogs subsequently developed local recurrence.^52^ Postoperatively, two dogs had a mild weightbearing lameness, one was intermittently non‐weightbearing, and one was non‐weightbearing.^52^ The latter two dogs were most lame of the 11 due to late‐stage fractures of the radius adjacent to the surgical site.^52^ That report did not document any functional outcome differences among the three dogs whose styloid process was removed compared to those whose styloid was spared.^52^ Additionally, the survival time of dogs undergoing ulnar ostectomy were not significantly different than those who underwent limb amputation.^52^ One study included 16 dogs that underwent ulnar ostectomy due to ulnar bone tumors.^53^ Three of these dogs experienced a biomechanical complication (19%): transient carpal instability in one, hemicerclage implant failure in two, and pain and swelling in the surgical site of one dog.^53^ Hemicerclage was elected in 4/16 dogs due to transection of interosseous ligament,^53^ which can lead to radial head luxation. Similarly to the previously discussed report, no adverse effects were reported due to removal of the styloid process.^53^

### 3.3 Humerus

Cortical allografting for comminuted humeral fractures was reported in one study including two cases of humeral fractures.^75^ In one study, 17 dogs with OSA received a cortical allograft at the proximal humerus, requiring scapulohumeral arthrodesis.^85^ Complications were common: infection, local recurrence, fracture the distal humerus, plate loosening in the humerus, and fracture of the allograft.^85^ Seven dogs (41%) underwent limb amputation due to complications.^85^ No biomechanical failures were observed in cases where double‐plating was performed.^85^ Postoperative limb function was poor in 10 dogs, fair in five, and excellent in two.^85^ Based on these results, it has been proposed that limb‐sparing at the proximal humerus should not be performed using cortical allografting.^85^

Intercalary bone grafts for the humerus appear to have good functional outcomes with excellent limb function postoperatively.^73^ The two humeral cases had diaphyseal neoplasia and therefore did not require a scapulohumeral arthrodesis.^73^

The use of irradiated autografts has been described at the humerus in five clinical cases.^37^ Four dogs developed a fracture of the autograft, warranting revision surgery (4 cases) or amputation (2 cases).^37^ The two cases that were amputated also had implant infections.^37^ One of the dogs who underwent revision surgery but not amputation experienced local recurrence of tumor.^37^ The median survival time for the humerus cases in this report is 298 days, and two cases were alive at the time of publication having survived greater than 1300 days.^37^ The other three cases succumbed due to metastatic disease.^37^

Frozen humeral autograft has been described in one dog with humeral OSA.^43^ The dog was alive and had subjectively good limb function 40 months postoperatively and had no evidence of metastatic disease but had a lung tumor excised which was a spindle cell sarcoma.^43^

### 3.4 Femur, coxofemoral joint and stifle joint

Cortical allografts for limb‐sparing at the femur due to comminuted fractures has been reported.^75^, ^86^, ^87^, ^88^, ^89^ In one report, dogs with comminuted femoral fractures experienced a high complication rate.^86^ In a subsequent case series of three dogs with comminuted femoral fractures treated with cortical allograft, all cases achieved normal ambulation postoperatively as reported by owners.^87^

One report included two dogs with distal femur OSA that were treated with a femoral cortical allograft.^67^ No infections were reported.^67^ Both dogs had poor limb function postoperatively attributed to the stifle arthordesis.^67^ One dog who had a necropsy was found to have microscopic local recurrence.^67^ Another study concluded that stifle arthrodesis can lead to severe postoperative lameness.^66^

Limb‐sparing surgery to address OSA of the proximal femur in a dog has been previously reported.^90^ The surgery used a combination of a cortical allograft and a cemented total hip replacement using a custom extra‐long stem femoral endoprosthesis. Of note, the surgeons were able to reattach the tendinous insertions of the gluteal muscles, internal hip rotators, vastus lateralis and iliopsoas using the allogenic donor tendons which were maintained during allograft harvesting.^90^ Limb function was deemed excellent by authors up until 270 days postoperatively when an implant luxation occurred.^90^ Prior to this, aseptic loosening of the femoral graft was noted which required surgical revision with a plate.^90^

A dog with femoral head fibrosarcoma was treated with a cemented total hip arthroplasty (THA).^91^ In that case, a previous biopsy had failed to identify neoplasia in a lytic lesion and therefore a THA was performed.^91^

One case report described surgical limb‐sparing of the canine coxofemoral joint due to histiocytic sarcoma.^92^ A partial hemipelvectomy, paired with a proximal femur ostectomy was performed and, subsequently, a custom‐designed titanium hemipelvic and proximal femoral endoprostheses which was screwed and cemented into the patient's remaining hemipelvis and femur respectively was done.^92^ Postoperatively, the dog experienced transient neuropraxia which resolved; otherwise, no biomechanical failures or infection were observed.^92^ The dog had subjectively good, pain‐free use of the limb 14 months postoperatively.^92^

Limb‐sparing of one dog's pelvic limb with medial femoral condylar nonunion has been reported using a custom‐designed total knee replacement.^93^ The dog was weightbearing the same day postoperatively and had resumed normal activities (hunting) 3 months postoperatively.^93^

The first reported case of the use of a frozen autograft was in a dog with distal femur OSA in which the affected segment of femur was transected proximally, treated with liquid nitrogen while still attached distally, soaked in distilled water to reach room temperature, and implanted back into the patient with additional stabilization with a plate.^41^ The dog had an amputation 180 days postoperatively due to local recurrence.^41^

Use of irradiated tumoral autograft has been applied clinically to the femur of one dog with OSA. No complications were reported and the dog survived for 189 days with local recurrence detected at 145 days followed by detection of metastatic disease.

### 3.5 Tibia

Five cases of tibial comminuted fractures treated with cortical allografting have been reported.^75^, ^86^ One report on cortical allografting included one dog with distal tibia OSA and one dog with proximal tibia OSA.^67^ The dog with proximal tibia OSA had poor limb function attributed to the stifle arthrodesis that was required for the allograft.^67^ Poor limb function was also reported in the single case of proximal tibia OSA in another study in which a cortical allograft was used in addition to adjuvant chemotherapy.^66^ In the study on intercalary bone grating, none of the three dogs treated at the tibia experienced implant failure and all had good to excellent functional outcomes.^73^ However, as previously pointed out, intercalary bone grafting does not involve arthrodesis.

Patient‐specific 3D‐printed endoprostheses have been reported for the distal tibia in two dogs and proximal tibia in one dog with bone neoplasia.^16^, ^80^ Pantarsal arthrodesis was performed in the dogs with distal tibial tumors and stifle arthrodesis was performed in the dog with the proximal tibial tumor. In one dog with a distal tibial tumor, a significant lameness persisted.^80^ The implant also failed 280 days postoperatively.^80^ The other dog with distal tibial tumor developed an infection and the limb was amputated.^16^ The dog with proximal tibial tumor only had a two‐week follow‐up reported.^16^

The earliest report of veterinary BTO for limb‐sparing was one canine case to treat an infected non‐union of the tibia at previous fracture site.^21^ BTO has also been performed for the distal tibial, and tibial diaphyseal sites for neoplasia and fracture.^23^, ^27^

Use of a frozen autograft for the tibia has been reported in one case—a chondrosarcoma of the proximal tibia treated with cryotherapy.^42^ In that case, the proximal tibia was resected to be treated, reimplanted, and stabilized with a plate that led to a stifle arthrodesis.^42^ No complications were reported.^42^ The only abnormality was the increased circumduction of that limb when walking due to the stifle arthrodesis.^42^

The first report of ITAP in dogs was of one dog who was victim to animal abuse and had both pelvic limbs partially amputated at the level of the distal tibia.^8^ A custom‐made combined endo‐exoprostheses were implanted in the remaining tibias.^8^ Although revision surgery was needed, the dog was reported to be ambulating well on both pelvic limbs 26 months after initial surgery.^8^ A subsequent case of ITAP with 3D printing for customized implant fabrication for tibial neoplasia has been reported.^9^ The dog had subjectively good limb function at 8 months postoperatively but soon after was diagnosed with hypertrophic osteopathy and euthanized due to presumptive metastatic disease.^9^

The use of irradiated autografts has been described at the tibia in six clinical cases.^37^ Two cases had no complications, three had fracture and implant failure requiring revision (*n* = 2) or amputation (*n* = 2, 1 dog had revision prior to amputation).^37^ One case experienced local infection requiring antibiotic therapy.^37^ Median survival time for the tibial sites in that report was 263 days.^37^ Local recurrence occurred in1 case and this dog also developed metastatic disease.^37^ Causes of death were metastatic disease in three cases, metastatic disease from a different neoplasia in one case, surgery failure in one case, and unspecified pain that was non‐responsive to therapy in one case.^37^

### 3.6 Scapula

In one study, one dog underwent total scapulectomy and three dogs underwent partial scapulectomy.^45^ Complete (or total) and partial scapulectomy has been reported with generally good to excellent limb function.^45^, ^46^, ^47^

The most extensive study on scapulectomy as a limb‐sparing approach included 42 dogs with scapular tumors.^49^ Dogs in that report underwent either partial scapulectomy, subtotal scapulectomy with preservation of the glenoid, or total scapulectomy.^49^ Acute complications were seroma formation, dehiscence, and intermittent weightbearing lameness.^49^ Chronic complications reported were: seroma formation >1 month postoperatively, pneumothorax, and long‐term lameness.^49^ Within 1 month postoperatively, limb function was subjectively evaluated in 41 dogs as poor in eight, fair in 17, good in 12, and excellent in four.^49^ Information on limb function >3 months postoperatively was only available for nine dogs in which one had good use of the limb and the others excellent use. The authors found that the amount of scapula removed was not associated with limb use status; however, they did find a significant negative association between patient bodyweight and postoperative limb use within 2 weeks of surgery.^49^

In addition to scapulectomy, frozen scapular autograft has been described in one dog with scapular OSA.^43^ The dog was alive and had subjectively good limb function 12 months postoperatively but had been documented to have metastatic disease at that time.^43^

## 4 DISCUSSION

The most common indication to perform limb sparing is primary neoplasia of bone in the appendicular skeleton, with OSA being by far the most common in dogs. There are many limb sparing techniques that are possible for a dog as has been described in the present review. After determining if a dog is a candidate for limb‐sparing (see indications and contraindications above), there are multiple factors to consider when deciding what technique to use for limb sparing. First is the assessment of the equipment and resources available to the surgeon. For example, does the surgeon have access to radiation therapy? If not, then all of the techniques that utilize radiation would be eliminated as an option. Availability of the required specialized equipment also affects the feasibility to perform pasteurization, microwave ablation, cryoablation, personalized 3D‐printed endoprostheses and microvascular transfer of autografts. Cortical allografts either require the maintenance of a bone bank, which is costly and time consuming, or purchasing a cortical allograft from a commercial source. Another factor is the skillset of the surgeon, that is, is the surgeon skilled in microvascular surgery (even if an operating microscope and instruments for microvascular surgery are available), or is the surgeon comfortable applying a ring fixator for BTO? Another consideration for BTO is whether there is the ability for the surgical team to be available to address concerns with the fixator as soon as they arise. This is a critical consideration for BTO since mechanical issues with the fixator that disrupt the distraction schedule can cause the transport segment to fuse prematurely or in an abnormal/undesired position. If the transport segment were to fuse, it would not be possible to move the bone segment anymore unless a more invasive surgery is performed to create another transport segment.

In addition to the equipment and resource considerations, the anatomic site is an important factor as well. For example, BTO would not be feasible for the proximal humerus. There can be tumor factors to consider: for URT and LMT, the tumor in the distal radius cannot invade into the distal ulna. Lastly, there are factors related to the owner to take into account: is the owner prepared to be responsible for daily distraction of the transport segment for BTO as prescribed, or does the owner have religious or cultural beliefs that preclude the use of an allograft? Given the lack of data comparing the different techniques and their clinical outcomes in veterinary medicine, including risks of complications, and the paucity of data for many techniques reported in this review where many techniques are often reported in a single case or only a handful of cases, the surgeon's preference finally becomes a significant factor. Table 3 summarizes numerous factors to consider for the different techniques. With the predominance of single case reports for several techniques at several anatomic sites (such as frozen tibial autografts, limb‐sparing secondary to pelvic or coxofemoral tumors as examples), it remains unclear whether there is comparitively less literature for such techniques and at these sites due to the reduced frequency of pathology warranting limb‐sparing techniques at these sites. Alternatively, it could be the case that these sites and/or techniques are more complex, difficult to perform, or have sufficiently high‐risk profile that surgeons do not recommend to clients. Lastly, such as in the case of cryotherapy for autografts or for customized implants, the equipment needed to facilitate techniques at these sites may be inaccessible for most surgeons. The lack of robust outcome information for a larger sample size for the majority of techniques makes it challenging to discuss with clients what the potential long‐term outcome and complications of the limb‐sparing could be.

**TABLE 3:** Factors to consider in choosing a limb sparing technique.

Surgical technique	Factors to consider
Cortical allograft	Access to/maintenance of a bone bank Owner's religious or cultural beliefs
Endoprostheses (metal, off‐the‐self)	Currently available implants do not allow for reliable bone ingrowth into the endoprosthesis. Therefore, plate and screws will need to be relied upon for the remainder of the dog's life Currently only described for the distal radius but could potentially be applied at other anatomic sites
Endoprostheses (metal and bioreplaceable, 3D‐printed, patient‐specific)	Feasibility to perform CT Expertise to design a personalized implant and ability to 3D‐print with the proper material
Endoprostheses (bioreplaceable, 3D‐printed, non‐patient‐specifc scaffolds)	Availability of scaffold made of an appropriate material
Bone transport osteogenesis	Surgeon who can/is comfortable with applying a ring fixator Surgical team available 24/7 for concerns with the construct Currently only described for the distal radius and distal tibia anatomic sites Owner compliance with distraction for every 6 h for months until distraction course is completed
Heterotopic autograft non‐vascular	Morbidity at the donor site Autograft may not integrate well in the new site, potentially increasing risks of complications compared to vascular grafts
Heterotopic autograft, microvascular transfer	Requires special equipment: operating microscope and microvascular instruments Surgeon with skills in microvascular surgery Morbidity at the donor site
Heterotopic autograft, ulnar rollover transposition	Tumor in the radius cannot invade distal ulna
Lateral manus translation	Tumor in the radius cannot invade distal ulna Equipment to facilitate the surgery and familiarity with string‐of‐pearls plating
Orthotopic autografts: Intraoperative radiation therapy Pasteurization Cryotherapy Microwave ablation	Availability of the specialized equipment required to perform the surgery (which will vary by technique)

Abbreviations: 3D, three‐dimensional; CT, computed tomography.

There are three main and most common complications associated with limb sparing: infection, biomechanical complications, and local recurrence of tumor. Current techniques of limb sparing have a relatively high rate of complications. In one study, 96% of dogs that had limb sparing of the distal radius performed using a metal implant had a complication.^11^ Factors that have been proposed to contribute to or cause the high complication rate with surgical limb‐sparing are: (1) extensive surgery in an area where there is poor soft tissue coverage (for some anatomic sites such as distal radius and distal tibia), (2) use of large non‐viable grafts with some techniques (e.g., cortical allograft, irradiated graft, cryotherapy), (3) use of large metallic implants with most techniques where implants are not amendable to being removed (large plates, many screws, large metallic endoprosthesis), (4) the patient will be repeatedly immune‐suppressed with chemotherapy in instances where the pathology was osteosarcoma, (5) for dogs with osteosarcoma, the tumor can lead to local immune suppression, and (6) most techniques lead to arthrodesis and some joints with an arthrodesis lead to poor limb function and potentially higher risk of biomechanical complications. The factors that lead to complications will therefore vary by anatomic site, the reason for limb spare surgery, and surgical technique. Some complications are severe enough and non‐resolvable to where it leads to an amputation of the limb,^76^ which is what the pet owner and veterinary team were trying to initially avoid. Most research and advances made in limb‐sparing in dogs are aimed at decreasing the risk of complications.

Infection remains the most common complication and arguably one of the most frustrating to treat. In one study, 16% of dogs that had a limb spare surgery eventually had the limb amputated.^76^ The most common reason for performing an amputation after limb spare surgery was infection, where 39% of amputations were performed because of an infection and for an additional 17%, infection was contributing to the complications leading to the amputation.^76^ In the quest to improve the outcome of limb sparing, better treatments for infections is an important dimension to keep pursuing. As infections become more common with multidrug resistant bacteria (author's observation, no published data in canine limb sparing), novel therapies are required. One promising avenue is the use of activated mesenchymal stromal cell therapy. Although a single care report, activated mesenchymal stromal cell therapy led to the successful treatment of dog with a limb spare with a multidrug resistant bacterial infection.^94^

As previously mentioned, dogs with an infection after limb sparing have on average a longer survival time.^74^, ^76^, ^77^, ^95^ This finding has also been reported in people with deep infections after surgical limb sparing for osteosarcoma.^96^ Interestingly, dogs that get an infection with an amputation do not have a survival advantage, which suggests that the intensity and/or duration of infection are important factors in prolonging survival.^97^ The current hypothesis for the longer survival provided by the infection is that the immune system becomes better at recognizing and destroying the tumor cells. A mouse model of OSA examined the effects of infection on tumor angiogenesis and innate immunity and demonstrated that chronic localized bacterial infection could elicit significant systemic antitumor activity dependent on natural killer cells and macrophages.^98^ In another study, canine macrophages exhibited increased IFN‐γ and TNF‐α cytokine secretion, decreased TGF‐β cytokine secretion, increased mRNA expression of TNF‐α and increased surface receptor expression of CD80 when cocultured with osteosarcoma and Staphylococcus aureus, suggesting that infection upregulates the inflammatory immune response to counteract osteosarcoma‐induced immune suppression.^99^

Successful limb sparing with minimal risk of complication remains a tremendous challenge in clinical practice today. Clinicians ultimately aim to restore the body to its original healthy state or as close to this as is realistically achievable for that individual patient. For limb sparing in veterinary medicine, this would mean restoring the animal's own viable, healthy bone without any implants or foreign materials remaining in the body in the most ideal scenario. A multidisciplinary approach will be necessary to achieve this goal with OSA. The best combination of modalities remains to be determined and likely will involve modalities that are only recently emerging such as bioengineering, immunotherapy, and others that may not exist yet. Continued efforts in this area are integral to improving the outcomes and quality‐of‐life of dogs affected by bone tumors or severe fractures.

While amputation can lead to behavioral changes according to the dogs' owners,^2^, ^4^ there is currently no data addressing this question for limb sparing. The goal with limb sparing is that by restoring the function of the limb to as close‐as‐normal and decreasing the rate of complications with new advancements, behavioral changes postoperatively would be minimized. Anecdotally, from one of the author's personal experience performing limb‐sparing surgeries, owners of dogs with a successful limb sparing surgery report a high quality of life, which could presumably imply return to, or close to, normal behavior.

## AUTHOR CONTRIBUTIONS

Altwal J, DVM: Conceptualization, writing original draft, writing – review and editing. Séguin B, DVM, MS, DACVS: Supervision, writing original draft, writing – review and editing.

## CONFLICT OF INTEREST STATEMENT

The authors have no conflict of interest, disclaimers, or source of support to disclose.
